# Relationships between Paraspinal Muscle Activity and Lumbar Inter-Vertebral Range of Motion

**DOI:** 10.3390/healthcare4010004

**Published:** 2016-01-05

**Authors:** Alister du Rose, Alan Breen

**Affiliations:** 1 Institute for Musculoskeletal Research and Clinical Implementation, Anglo-European College of Chiropractic, Parkwood Road, Bournemouth BH5 2DF, UK; imrci.ABreen@aecc.ac.uk; 2 Faculty of Science and Technology, Bournemouth University, Fern Barrow, Poole BH12 5B, UK

**Keywords:** spine kinematics, fluoroscopy, surface electromyography, reliability, agreement

## Abstract

Control of the lumbar spine requires contributions from both the active and passive sub-systems. Identifying interactions between these systems may provide insight into the mechanisms of low back pain. However, as a first step it is important to investigate what is normal. The purpose of this study was to explore the relationships between the lumbar inter-vertebral range of motion and paraspinal muscle activity during weight-bearing flexion in healthy controls using quantitative fluoroscopy (QF) and surface electromyography (sEMG). Contemporaneous lumbar sEMG and QF motion sequences were recorded during controlled active flexion of 60° using electrodes placed over Longissimus thoracis pars thoracis (TES), Longissimus thoracis pars lumborum (LES), and Multifidus (LMU). Normalised root mean square (RMS) sEMG amplitude data were averaged over five epochs, and the change in amplitude between epochs was calculated. The sEMG ratios of LMU/LES LMU/TES and LES/TES were also determined. QF was used to measure the maximum inter-vertebral range of motion from L2-S1, and correlation coefficients were calculated between sEMG amplitude variables and these measurements. Intra- and inter-session sEMG amplitude repeatability was also assessed for all three paraspinal muscles. The sEMG amplitude measurements were highly repeatable, and sEMG amplitude changes correlated significantly with L4-5 and L5-S1 IV-RoMmax (r = −0.47 to 0.59). The sEMG amplitude ratio of LES/TES also correlated with L4-L5 IV-RoMmax (r = −0.53). The relationships found may be important when considering rehabilitation for low back pain.

## 1. Introduction

Optimal control of the spine during voluntary trunk bending requires fine-tuned coordination of numerous trunk muscles [1]. This dynamic control is believed to be modulated by communication between three sub-systems, the passive (vertebrae, discs, and ligaments), the active (muscles and tendons), and the control (central nervous system and nerves) systems [2,3]. Investigating the interplay between sub-systems however is difficult, as the spine is a complex structure; and a hidden kinematic chain. Several different technologies are therefore typically required, each with their own limitations.

In order to directly investigate the passive and active sub-systems of the spine, there have been many efforts to concurrently measure spinal kinematics and muscle activity [4,5,6,7,8,9,10,11,12]. The majority of these studies have used surface electromyography combined with skin surface kinematic measurement techniques such as Fastrak [8,13], Isotrak [9,11,12], or cameras [4,5,7]. These are limited to the investigation of gross spinal motion. To include segmental data usually requires invasive techniques such as the surgical insertion of intra-osseous pins. In this way Kaigle *et al*. (1998) investigated the reduction in lumbar muscular activity during full flexion (flexion relaxation) and spinal kinematics at an inter-vertebral level [10]. However, typically only single motion segments were considered, and EMG was also only recorded from one level (e.g., lumbar longissimus thoracis) [10].

### 1.1. Contemporaneous Monitoring of Inter-Vertebral Passive and Motor Control Systems

Study of the integrated function of the joints and muscles of the spine requires contemporaneous multi-level kinematic and electromyographic monitoring throughout the motion. This is necessary to incorporate timing, magnitude, and segmentation in the two systems to characterise control. Multi-level surface electromyography fulfils these requirements for motor control and quantitative fluoroscopy measures a range of continuous inter-vertebral motion variables [14]. Contemporaneous recording of these measures therefore provides an integrated assessment of the passive and active systems of the spine, and it is proposed that this may be useful when assessing patients with low back pain (LBP) [4,15]. This study therefore deployed quantitative fluoroscopy (QF), and surface electromyography (sEMG) of the lumbar spine together for the first time. The study investigated the biomechanics of the lumbar spine in a healthy control population in order to potentially better understand the significance of biomechanical changes in LBP populations.

### 1.2. Variable Selection

In order to investigate relationships between segmental kinematics and local muscle activity, suitable variables from each must be identified. While responses to perturbation [16], and the flexion relaxation phenomenon (an absence of paraspinal muscle activity during full sagittal flexion (FRP)) have been investigated [17,18], few studies have included sEMG amplitude changes throughout the cycle, be they increases or decreases. This study therefore addressed these parameters. QF measures continuous intervertebral rotation and translation in the coronal and sagittal planes during weight-bearing or recumbent motion and can also extrapolate the instant axis of rotation (IAR) and rotational range attainment rate from this. However, the need to also compare intervertebral range of motion (IV-RoM) with sEMG in the present studies, dictates the need for continuous motion information. Therefore IAR rotation and attainment rate were not likely to be so useful. In addition, the small ranges of translation make this measure unsuitable for numerical comparisons, leaving maximum rotational motion as the preferred measure.

To investigate the relationships between lumbar muscle activity and inter-vertebral restraint during bending requires access to the maximum IV-RoM (IV-RoMmax). Continuous intervertebral rotation data allows both temporal comparisons with other variables and the actual maximum IV-RoM (IV-RoMmax), rather than IV-RoM at the limit of voluntary trunk bending, to be extracted. Recording in the standing orientation allows these comparisons.

### 1.3. Enhanced Functional Assessment

Sanchez-Zuriaga *et al*. (2015) suggest that there are only subtle differences between various low back patient groups and healthy controls in terms of paraspinal muscle activity and regional lumbar movement [4]. This means that either muscle activity has no effect on the range of motion, or that we are missing the detail of what is happening at individual levels. For example it may be that whereas there is an increase in paraspinal activity in recurrent LBP patients during flexion, but no difference in RoM, the share of RoM may have shifted between levels at different stages in the motion. The primary role of the paraspinal muscle during flexion is to resist inter-vertebral motion [19] and so it may be that the motion is restricted at a specific level, and compensated for elsewhere, be this at other lumbar levels, or in the thoracic spine or pelvis. It is essential therefore, when attempting to understand the relationships between functional impairments and LBP that specific inter-vertebral levels are assessed both in terms of kinematics and associated muscle activity.

### 1.4. Repeatability

The development of QF techniques has seen its use in LBP research become more common [20,21,22]. IV-RoM has been the most common QF measure of inter-vertebral motion [22,23,24], where it has been shown to be accurate and reliable [22,25]. It is known however that sEMG recordings, by contrast, are inherently variable [26,27]. Therefore, a sub-study was conducted to assess the intra and inter-session repeatability (reliability and agreement) of the mean normalised root mean square (RMS) sEMG amplitude recordings from the entire flexion and return cycle.

### 1.5. Aim of the Study

The purpose of this study was to quantify the relationships between IV-RoMmax during flexion of the lumbar spine with the accompanying paraspinal muscle activity.

### 1.6. Specific Objectives

To determine the inter- and intra-session reliability and agreement of normalised sEMG amplitudes during weight-bearing sagittal flexion and return.

- To determine whether ratios of inter-level lumbar paraspinal sEMG amplitudes are related to the IV-RoMmax at lumbar inter-vertebral levels.
- To determine whether changes in sEMG amplitudes during different phases of the forward bending cycle are related to IV-RoMmax at lumbar inter-vertebral levels.

## 2. Experimental Section

### 2.1. Participants

The eligibility criteria for the study are shown in Table 1. Twenty male participants from the Anglo-European College of Chiropractic (AECC) student population were recruited. National Research Ethics Service (NRES) approval was gained for the study (Bristol 10/H0106/65) and written informed consent was obtained from all participants prior to data collection. The QF and sEMG data collection was conducted concurrently. In order to minimise the potential impact of variations in parameters such as soft tissue thickness (STT) and spinal degeneration (e.g., reduced disc heights), recruitment was restricted to young adult males.

**Table 1:** Eligibility criteria.

Inclusion	Exclusion
Males aged 20–40 years	Poor understanding of English
Able to understand written information	Having treatment for osteoporosis
Willing to participate and able to give informed consent	Recent abdominal or pelvic surgery
Consent to GP being informed	Previous lumbar spine surgery
BMI < 30	BMI > 30
No history of low back pain that prevented normal activity for at least one day in the previous year	Any medical radiation exposure in the past year or exposure in the past two years with a dose greater than 8mSv
	Current involvement in any other research study

### 2.2. Kinematic Data Collection and Processing (Quantitative Fluoroscopy)

**Figure 1:**
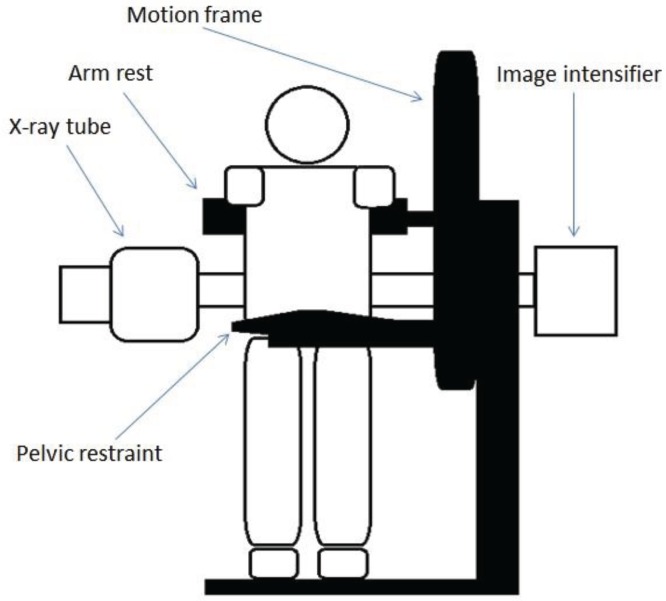
Fluoroscope and motion frame set-up.

Lumbar spine fluoroscopic images were collected at 15 Hz using a Siemens Arcadis Avantic VC10A digital fluoroscope (CE0123) and an upright motion frame, which stabilised participants and guided their bending motion. Participants were asked to stand with their right side against the motion frame (Figure 1), and follow a rotating arm rest which guided them through a range of 60° of forward flexion and a return to upright during continuous fluoroscopic imaging over a period of 20 seconds. A range of 60° was selected on the basis that the lumbar spine has an overall range of 80° (Flexion and extension components) [28]. The motion frame apparatus could be fully adjusted in accordance with the participant’s stature, and the central ray was positioned at L4 to ensure that all vertebrae (L2-S1) were included in the image field. 

Before image acquisition commenced, participants were taken in 20° increments through to the full 60° to ensure that they were able to tolerate the motion. The movement of the motion frame was recorded by electronic feedback from its motor drive and synchronised with the fluoroscopic imaging. To avoid bending at the hip joints, the pelvis was stabilised using a belt secured around the anterior superior iliac spine and secured to a bracing pad placed against the lower sacral segments. A lead apron was worn to shield the gonads. 

Flexion and return sequences were then transferred to a desktop computer for analysis using bespoke image processing codes written in Matlab (The Mathworks, Cambridge) [14]. The vertebral outlines from L2-S1 in the first image in each sequence were manually marked with an electronic template using the screen cursor. This process was repeated five times for each sequence and the results averaged to increase precision. In each subsequent image frame the software programme automatically tracked each vertebra, producing a continuous measurement of its movement throughout the bending sequence [14]. Template tracking was checked visually via video playback to ensure the templates maintained the correct alignment throughout the sequence.

The data extracted comprised the continuous inter-vertebral angle in flexion and the IV-RoMmax. IV-RoMmax for each inter-vertebral level (L2-3, L3-4, L4-5 and L5-S1) was calculated as the maximum angular range reached at any point throughout the 60° trunk flexion and return cycle.

### 2.3. Electromyography

**Figure 2:**
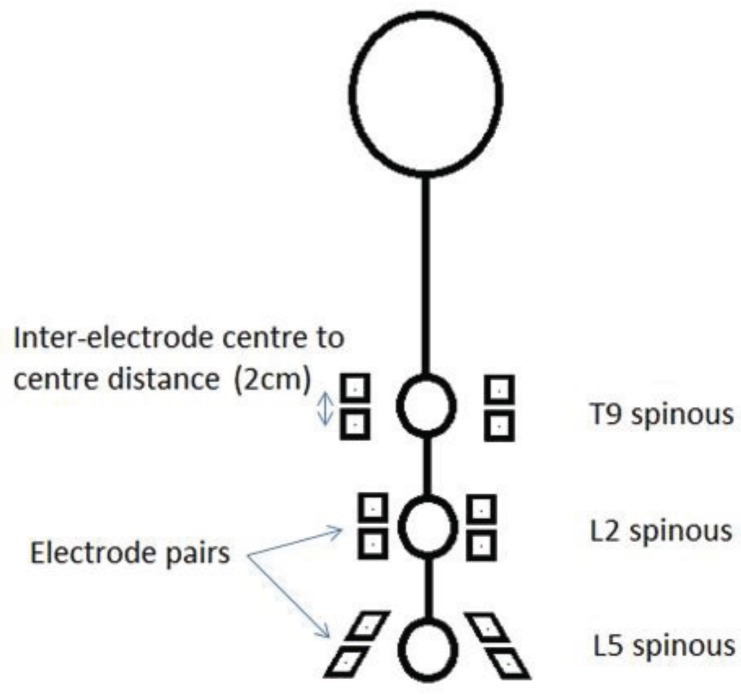
Electrode positioning sites. (Note: T9 spinous refers to the spinous process of the ninth thoracic vertebra, L2 to the second lumbar vertebra and L5 to the fifth lumbar vertebra.)

Prior to the commencement of data collection, participants lay prone in order for 12 electrode sites to be marked on their backs with a skin pencil. In preparation for this, the skin over their lower backs was prepared for sEMG electrode application by light abrasion, cleaning with an alcohol swab, and when necessary, shaving of the area. Disposable pre-gelled self-adhesive Ag-AgCl electrodes were then applied over three bilateral muscle groups with a 20 mm centre-to-centre inter-electrode distance as follows: Thoracic erector spinae (TES) (5 cm lateral to the T9 spinous process) [12,29], the lumbar erector spinae (LES), and lumbar multifidus (LMU) (2 cm lateral to the L2 and L5 spinous processes) [18,30] whilst the participant was in slight flexion (Figure 2).

Although cross talk from multiple muscles will inevitably contribute to the signal recorded at each electrode site, cross-sections of the spine at each electrode site showed that the muscles that will predominate at T9 (TES) and L2 (LES) is longissimus thoracis, and at L5 (LMU) multifidus [31]. Three Biopac wireless transmitters (Bionomadix Dual Channel Wireless EMG) were then placed on the lower back attached by self-adhesive Velcro pads. There was no significant difference between the normalised mean sEMG amplitudes recorded over left and right sides during the flexion and return cycle. Therefore, an average of the mean amplitudes from both sides was used for all analysis [15].

### 2.4. Electrode Positioning Accuracy

**Figure 3:**
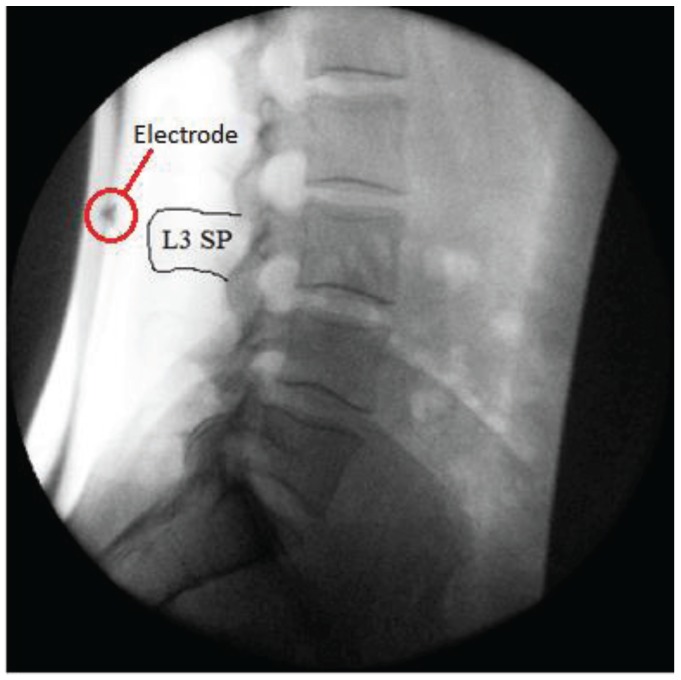
An electrode placed over the spinous process of L3.

Electrode application accuracy is dependent on the subjective identification of bony anatomical landmarks, and current methods used are therefore limited by human subjectivity and variation in individual anatomy [32,33,34,35]. It has been suggested however that accuracy can be improved significantly when techniques are combined [36]. This investigation was integrated into a larger ongoing normative database study, which required recumbent QF imaging before weight-bearing imaging commenced. In order to improve electrode positioning accuracy, an electrode was placed over the spinous process of L3 during the recumbent protocol. This provided an improved anatomical reference point for the application of the electrodes (Figure 3). 

### 2.5. The sEMG Equipment

The sEMG signal data were recorded at a sampling rate of 2000 Hz using a common-mode rejection ratio (CMRR) of 110 dB and an input impedance of 1000 MOhms. 

The six signals were band pass filtered at 10–500 Hz and full wave rectified. The root mean square (RMS) amplitude was calculated for individual participant cycles and normalised during post-processing to sub-maximal voluntary contractions expressed as a percentage of the sMVC.

### 2.6. Reference Contraction

When data collection had been completed, and in order to provide a sub-maximal reference contraction (sMVC) [37], participants were asked to lie prone on a padded bench with their hands behind their head. They were then required to raise their torso off the couch and hold this position for five seconds whilst their legs and pelvis were stabilised. This process was repeated three times and the average sMVC was used as a reference. This technique was selected over a normalisation to a peak, primarily due to the even loading of the investigated muscle groups, but also to avoid the problem of variations in participant’s muscle activation patterns in order to produce the same movement. 

### 2.7. Synchronisation

**Figure 4:**
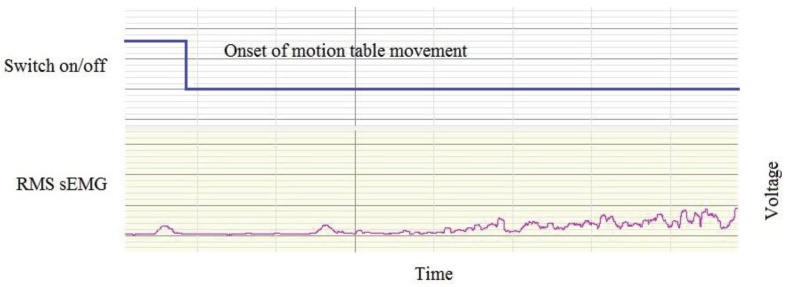
Synchronisation of the motion frame movement and sEMG recordings.

The QF motion frame controller recording and the sEMG data recording were co-ordinated using a trip switch attached to the motion arm of the frame. This registered a data point on the sEMG timeline (Figure 4).

### 2.8. The sEMG Amplitude Repeatability Study

A separate convenience sample of 10 participants was used for the sEMG amplitude intra- and inter-subject repeatability studies. These studies were done without QF imaging. The acquisition cycle was repeated four times (several minutes apart) at baseline and follow up. Intra-session results compared cycles 1 and 2 (of the four), whereas inter-session results were calculated as an average of the four mean (left and right) normalised amplitudes recorded over the cycle duration. All analysis was conducted by ADR.

### 2.9. Data Analysis

sEMG ratios [38,39] were calculated from the mean left-right normalised sEMG (RMS) amplitudes during the flexion phase only as follows, LMU/LES, LES/TES and LMU/TES. In order to calculate sEMG changes at different stages of the flexion cycle, the forward bending phase was divided into five epochs for each participant [15]. The change in mean sEMG between epochs was then calculated (e.g., the change during the early stage of flexion was calculated as (epoch 1–2) for each of TES, LES, and LMU). This was repeated to determine changes between all epochs at all levels.

All data were tested for normality using the Shapiro-Wilk test. Relationships between IV-RoMmax and sEMG ratios and changes from normally distributed data were analysed using the Pearson product-moment correlation coefficient, and non-normal data using the Spearman’s Rank Correlation. Significant relationships (*p* values < 0.05) were further analysed using simple linear regression. Intra-subject reliability and agreement of the mean normalised RMS sEMG amplitudes throughout the flexion and return cycle were assessed using intra-class correlations (ICC 3, 1) [40], and the standard error of measurement (SEM) respectively [41]. Statistical analysis was performed using IBM SPSS (version 21).

## 3. Results and Discussion

### 3.1. Results

Twenty males with no history of low back pain over the previous year consented to participate. Failed template tracking occurred in two participants’ sequences, and their QF and sEMG data were therefore discarded. The mean (SD) age, height, and body mass Index (BMI) were 27.6 years (4.4), 1.8 m (0.06), and 24 (2.2), respectively. Average radiographic exposure factors for the group were recorded as 79.7 kV SD (5.4) and 55.4 mA SD (3.4). The mean effective dose was calculated using ICRP103 conversion software PCXMC (Monte Carlo Simulation Package) [42], and was 0.143 mSv. A complete motion sequence of the lumbar spine therefore requires less radiation than a single traditional radiograph [14]. Mean normalised RMS sEMG during the flexion cycle ranged between 3% and 21% for the TES, 2% and 31% for the LES and 13% and 40% for the LMU. 

#### 3.1.1. Reliability and Agreement

Intra- and inter-session reliability and agreement for normalised muscle activity during the bending sequence was high for all muscle levels (Table 2). The highest ICC was for LMU intra-session (ICC = 0.990, 95% CI 0.961–0.998), and the lowest SEM was 0.5% for TES intra-session. The lowest ICC was for LES inter-session (ICC = 0.872, 95% CI 0.508–0.968) and the highest SEM was for LES inter-session (SEM = 3.9%).

**Table 2:** Intra- and inter-session reliability and agreement for normalised RMS sEMG amplitudes during the weight-bearing sagittal plane QF protocol (n = 10).

	Intra-Session ICC (3, 1) (95% CI)	Inter-Session ICC (3, 1) (95% CI)	Intra-Session SEM (%)	Inter-Session SEM (%)
TES	0.996 (0.986–0.999)	0.895 (0.606–0.974)	0.5	2.7
LES	0.984 (0.939–0.996)	0.872 (0.508–0.968)	1.2	3.9
LMU	0.990 (0.961–0.998)	0.974 (0.902–0.993)	1.4	2.8

#### 3.1.2. Correlations between Muscle Activity Changes and IV-RoMmax

A summary of all correlations between changes in muscle activity and IV-RoMmax is given in (Table 3). Significant correlations were only found with lower lumbar segmental motion (L4-5 and L5-S1). These were consistently of mid-level strength (r-values ranging from –0.48 to 0.59), and include inter-vertebral relationships with all three muscle levels. The results also demonstrate a number of correlations that approach significance; these did include relationships with motion at upper inter-vertebral lumbar levels (L2-3 and L3-4). 

All significant correlations were further analysed using simple linear regression. The effects of muscle activity changes on IV-RoMmax are shown in (Table 4). The table shows that r^2^ values range from 0.177 to 0.247.

**Table 3:** Correlations* between muscle activity changes (three groups, five epochs) and IV-RoMmax at all inter-vertebral levels (n = 18).

		Inter-Vertebral level			
Muscle activity change		L2-L3	L3-L4	L4-L5	L5-S1
TES epoch 1-2	r	**0.404**	0.316	−0.164	0.224
	*p*	**0.097**	0.201	0.516	0.371
TES epoch 2-3	r	0.083	−0.02	0.036	**−*0.477***
	*p*	0.743	0.938	0.888	***0.045***
TES epoch 3-4*	r	−0.059	−0.077	−0.171	**−0.434**
	*p*	0.817	0.760	0.496	**0.072**
TES epoch 4-5	r	−0.124	−0.194	−0.134	−0.103
	*p*	0.625	0.441	0.596	0.683
LES epoch 1-2*	r	−0.203	0.070	***0.595***	0.391
	*p*	0.418	0.782	***0.009***	0.108
LES epoch 2-3	r	−0.045	0.257	0.295	***0.497***
	*p*	0.86	0.303	0.234	***0.036***
LES epoch 3-4	r	−0.117	−0.118	0.211	0.266
	*p*	0.645	0.642	0.4	0.286
LES epoch 4-5*	r	0.228	0.215	−0.088	−0.055
	*p*	0.362	0.392	0.729	0.829
LMU epoch 1-2	r	0.14	0.334	0.314	−0.144
	*p*	0.58	0.176	0.204	0.567
LMU epoch 2-3*	r	0.021	0.062	0.317	0.139
	*p*	0.935	0.807	0.200	0.581
LMU epoch 3-4	r	−0.039	0.164	**0.455**	0.273
	*p*	0.877	0.517	**0.058**	0.272
LMU epoch 4-5	r	−0.159	0.067	**0.429**	***0.461***
	*p*	0.53	0.793	**0.076**	***0.027***

Significant correlations are highlighted in bold italic. Correlations that approach significance are highlighted in bold. * Indicates a row that includes non-parametric data and therefore a Spearman’s Rank Correlation was used. All other normally distributed data were analysed using the Pearson product-moment correlation coefficient. r = correlation co-efficient, *p* = *p*-value.

**Table 4:** Simple linear regression analysis: significant correlations.

Variable	Inter-Vertebral Level	r	*p*	r²
LMU Epoch 4-5	L5-S1	0.461	0.027	0.212
LES Epoch 2-3	L5-S1	0.497	0.036	0.247
TES Epoch 2-3	L5-S1	−0.477	0.045	0.227
LES Epoch 1-2*	L4-5	0.595	0.009	0.177

* Indicates a row that includes non-parametric data and therefore a Spearman’s Rank Correlation was used. All other normally distributed data was analysed using the Pearson product-moment correlation coefficient. r = correlation co-efficient, *p* = *p*-value and r^2^ = the co-efficient of determination.

#### 3.1.3. Correlations between sEMG Ratios and IV-RoMmax

**Figure 5:**
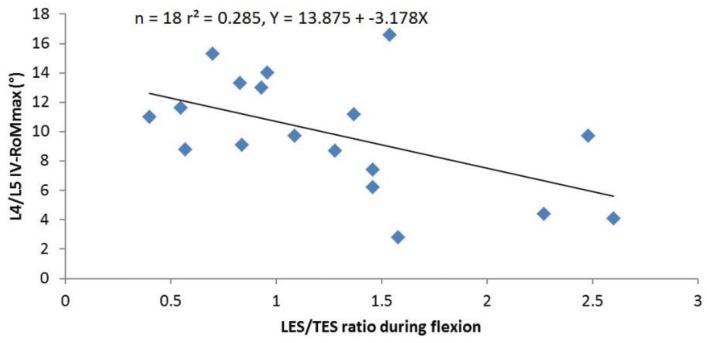
The relationship between the ratio of LES/TES and the IV-RoMmax at L4-5.

The correlations between sEMG ratios and IV-RoMmax at all inter-vertebral levels are shown in (Table 5). The only significant relationship was found between the ratio of LES/TES and the IV-RoMmax at L4-5, and is demonstrated by the scatter plot in (Figure 5). This plot highlights the negative correlation between the LES/TES ratio and L4-L5 IV-RoMmax, and shows that when the muscle activity of the LES increases relative to that of the TES, there is a decrease in the IV-RoMmax at L4-L5. The only other correlation to approach significance was between LMU/LES ratio and the IV-RoMmax at L5-S1 (r = 0.37, *p* = 0.13). 

**Table 5:** Correlations between muscle activity ratios and IV-RoMmax at all inter-vertebral levels (n = 18).

		Inter-Vertebral Level			
Ratio		L2-L3	L3-L4	L4-L5	L5-S1
LMU/TES	r	0.046	−0.013	−0.236	0.152
	p	0.856	0.958	0.345	0.548
LMU/LES	r	−0.209	0.04	0.263	0.37
	p	0.405	0.875	0.292	0.13
LES/TES	r	0.095	−0.217	−0.533	−0.242
	p	0.708	0.387	0.023	0.333

r = the Pearson product-moment correlation coefficient, *p* = *p*-value.

### 3.2. Discussion

#### 3.2.1. Reliability and Agreement

It is recommended that any procedures to be used in EMG studies should undergo reliability testing [43]. In this study, intra- and inter-session reliability and agreement was “substantial” for all muscle levels [40]. A common problem with sEMG studies is the great variability in their findings [44,45], therefore the high reliability shown in this study is reassuring. It is usual for a proportion of variability to be attributed to a lack of standardisation, and the method by which EMG variables are normalised [46]. The results however (Table 2) indicate that the standardisation of movement range, speed, and direction provided by the QF protocol may have played an important role in reducing the impact of variability resulting from these causes. It should be observed however that reliability and agreement was relatively poorer in the inter-session group, and of particular note was the increase in SEM for LES (3.9%). As muscle activity changes can be subtle during functional tasks, this may be a limitation for future inter-session studies.

#### 3.2.2. Changes in sEMG Amplitudes at Different Stages of the Flexion Cycle

**Figure 6:**
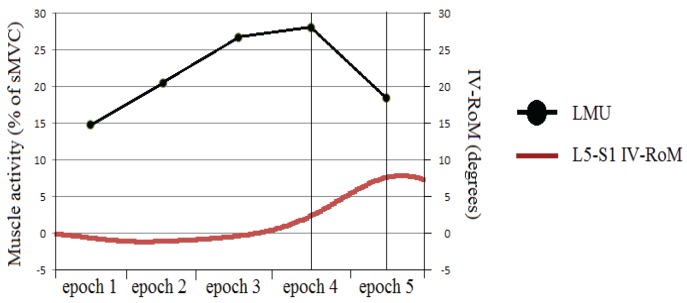
An example of LMU activity and lumbar IV-RoM during sagittal flexion.

The results demonstrate that changes in activity of TES, LES, and LMU at various stages of the forward bending cycle, can all be to some degree related to the IV-RoMmax at lower lumbar levels (L4-5 and L5-S1). It has been suggested that intersegmental forces maintain or decrease inter-vertebral motions [47,48], it would seem logical then that if the role of the posterior muscles is to resist sagittal flexion, in order for inter-vertebral movement to occur, there must be a deactivation of this supporting musculature. Figure 6 shows an example of how the muscles most local to the L5-S1 inter-vertebral segment (LMU) demonstrate a significant decrease in activity during the final stage of flexion in a healthy control subject. This corresponds with the phase lag [49] in the initiation of movement at the adjacent inter-vertebral level from the motion graphs. The larger the change in activity between epochs, (in this case deactivation in the final stages of the flexion cycle) the larger the IV-RoMmax at L5-S1. This is suggestive of a degree of localised control, however, the stabilisation of the pelvis in order to keep the spine in the image frame and avoid hip joint contributions to motion cannot be ruled out as possible external influences. This direct relationship between corresponding levels was not apparent between the LES and the upper inter-vertebral lumbar motion segments (Table 5), and may be suggestive of anatomically specific control at this level. However, the potential importance of LES and TES was also highlighted.

**Figure 7:**
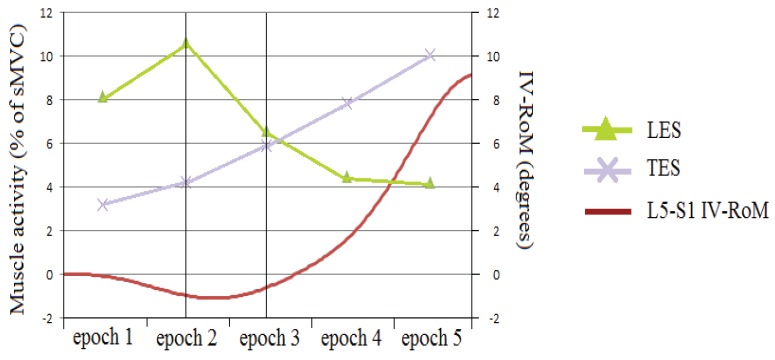
An example of LES and TES activity and L5-S1 IV-RoM during sagittal flexion (An example of a greater IV-RoMmax). Please note that the scales of both Y-axis are slightly different to those seen in Figure 8.

**Figure 8:**
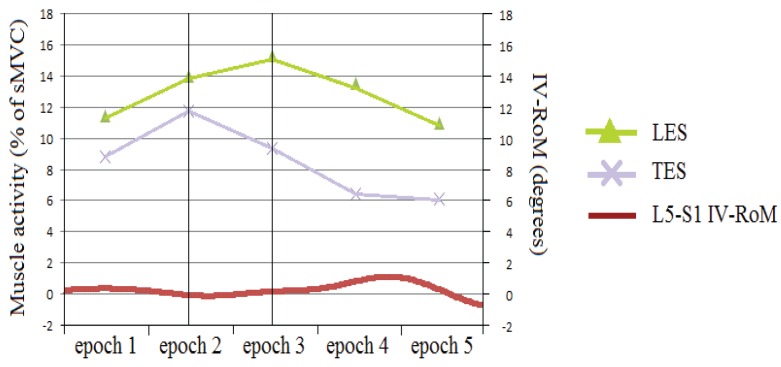
An example of LES and TES activity and L5-S1 IV-RoM during sagittal flexion (An example of a smaller IV-RoMmax). Please note that the scales of both Y-axis are slightly different to those seen in Figure 7.

Of particular interest is the apparent shift in effect between TES and LES on the IV-RoMmax of L5-S1 (Figure 7). As LES activity decreases between epochs 2 and 3 of the cycle (early mid stage) there is an associated increase in L5-S1 IV-RoMmax, whilst at the same stage of the cycle TES changes (decrease) are significantly associated with a decrease in L5-S1 IV-RoMmax (Figure 8). This indicates possible different roles for TES and LES in terms of the control of the range of motion at a distal motion segment. If there is more movement at L5-S1 there may be less activity of LES, more TES, and vice versa.

When considering the LES to be local (inter-segmental) and TES to be global (multi-segmental) [50], then these findings may have important clinical implications, as they raise the possibility of level specific stabilisation/control. Conflicting arguments have been put forward regarding the role of local and global muscles in spinal stability, Bergmark suggested that inter-segmental (local) muscles were the chief stabilisers [50], whereas Crisco and Panjabi concluded that the larger multi-segmental (global) muscles were more powerful [51]. In a study investigating the relative contribution of different trunk muscles to lumbar stability, Cholewicki and Van Vliet concluded that whilst inter-segmental and multi-segmental paraspinals had the greatest effect on stabilisation compared to other muscles (psoas and rectus abdominis), no distinction could be made between the two [52]. 

There are many correlations that approach significance (Table 3), and therefore future studies with a larger sample size may well reveal more statistically important relationships, potentially with upper lumbar inter-vertebral levels.

#### 3.2.3. The sEMG Ratios

Previous work has indicated a clear distinction between the kinematic behaviour of the upper and lower sections of the lumbar spine [53]. It was anticipated therefore that there may be relationships between the IV-RoMmax and the muscle activity ratio of LMU/LES. These were not evident, and suggest that the location of a motion segment within the spinal curvature, or the influence of passive structures (e.g., the strong iliolumbar ligament) may influence such interactions. The ratio of LES/TES however, did reveal a statistically significant negative relationship with the range of motion at L4-L5 (Figure 5 and Table 5). 

The ratio of lumbar erector spinae over thoracic erector spinae activity has been investigated in several previous studies [38,39,54,55,56]. In a musculoskeletal trunk model based on the EMG data collected from two healthy participants, Cholewicki and McGill suggested that the preferential recruitment of the LES over the TES may be a strategy to increase spinal stiffness [54]. A further study comparing the muscle recruitment patterns in healthy controls to those of LBP patients, found higher LES/TES ratios in the latter [38]. These results led to the conclusion that the differences found between groups were likely to be an adaptation designed to enhance spinal stability. This theory was further supported by Van Den Hoorn *et al*. (2012), who also demonstrated a significantly higher LES/TES ratio in LBP patients during gait [55]. 

Reeves *et al*. also investigated this muscle activation imbalance in varsity athletes, and while maintaining that there was indeed a relationship between muscle imbalance between levels and LBP, the authors also found that in some individuals with a history of LBP, TES activity could be dominant [39]. The authors contend that this may be explained by pathology, e.g., the CNS optimising activation to minimise compression, or by a difference in muscle fibre types between groups in order to compensate for fatigue related pain [39]. Crucially however, there is also the mention of the possibility of the patterns being the result of different types of posture or lordosis, and that further studies may account for this effect.

The results of this study highlight that the ratio of LES/TES can vary in a population with no long term history of low back pain, and would appear to relate to variations in inter-vertebral mechanics in such a population. It has been proposed that lumbar inter-segmental movement is also influenced by spinal morphology [57], but these results provide more level-specific detailed information, and it is apparent that different recruitment strategies are required in accordance with inter-vertebral range changes. A question frequently asked in this field of research is whether these strategies are a cause or a consequence of the related kinematics. 

It has been suggested that muscle imbalance between levels does not cause low back injury [39]. It is also suggested that imbalance is not necessarily tantamount to impairment. Therefore correcting muscle imbalance in patients should not be a priority. However if L4-L5 or L5-S1 are the segments of interest, or suspected levels of pain generation and movement at that level is considered to be part of the problem, then reducing the muscle imbalance may be of importance. 

In a LBP free population sample, it might be assumed that variations in muscle activity patterns do not represent adaptations to pain. However, that is not to say that particular activity patterns and thus kinematic behaviours may not be risk factors for future LBP episodes. It also questions the conclusions of studies that compare LBP population groups with healthy pain free controls, as muscle activity patterns may not be adaptations to the episode.

It is suggested that achieving sufficient stability is a moving target, and that no single muscle can therefore be considered the best stabiliser, as the most important muscle is transient dependent on the task [58]. The results provide a demonstration of this concept in action during the task of forward bending. Whilst effect sizes are small, inter-vertebral movements have been shown here to be influenced by muscle activity. It would seem that IV-RoMmax depends not only on the relative activation of multiple trunk muscles, but also other biomechanical variables, therefore, the next logical step may be to assess the importance of each. This will require multivariate analysis of larger population samples. If the relative value of each factor can be determined, then better informed decisions regarding model types and inputs may be possible. The diversity of muscle activation patterns within a “normal” sample highlights the problem of using limited participant numbers as a basis for systems models, whereas reductionist approaches are typically weakened by the limitations of the size of the effects of the selected variables. If the variables with the greatest influence on kinematics can be found, then the selective use of these variables in models and LBP/control studies may be beneficial. 

Finally, it is a limitation of this study that people with non-specific low back pain were not included, yet it would be important to know to what extent these relationships, which are consistent with maintaining appropriate restraint on vertebrae during bending, are disrupted in patients. If so, this would point to a potential route for patient stratification based on biomechanics. Such studies are now warranted. The study also only investigates a narrow population (*i.e*., young healthy male adults) and so the results are not generalizable to other groups. It is anticipated that variations in kinematic and morphological parameters that are associated with age related change and gender would also affect IV-RoMmax, and therefore also warrant further investigation. Future investigators may also wish to incorporate measurements such as thoracic kyphosis and pelvic incidence in order to gain insight into changes in kinematic behaviour beyond the lumbar spine.

## 4. Conclusions

This study found weak to moderate but significant correlations between both muscle activity changes and ratios and IV-RoMmax at various inter-vertebral levels. Of particular interest was the correlation between decreased LMU and increased IV-RoMmax at L5-S1 in the latter stages of flexion, the apparent co-dependency between LES and TES during early to mid-flexion, and the effect of the LES/TES ratio on the IV-RoMmax at L4-L5. These relationships, when combined with other influencing factors, may be important when specific inter-vertebral levels are considered to be sources of pain generation and when considering rehabilitative or surgical planning. Multivariate investigations in larger samples are warranted, potentially leading to longitudinal outcome studies in LBP groups.

## Abbreviations

The following abbreviations are used in this manuscript:

### Abbreviations

AB: Alan Breen
ADR: Alister du Rose
AECC: Anglo-European College of Chiropractic
BMI: Body Mass Index
CI: Confidence Interval
CMRR: Common Mode Rejection Ratio
CNS: Central Nervous System
EAC: European Academy of Chiropractic
EMG: Electromyography
FRP: Flexion relaxation phenomenon
GP: General Practitioner
IAR: Instant axis of rotation
ICC: Intraclass Correlation Coefficient
IV-RoM: Inter-vertebral Range of Motion
IV-RoMmax: Maximum Inter-vertebral Range of Motion
LBP: Low Back Pain
LES: Lumbar erector spinae
LMU: Lumbar multifidus
NRES: National Research Ethics Service
QF: Quantitative Fluoroscopy
RMS: Root Mean Square
RoM: Range of Motion
SD: Standard Deviation
SEM: Standard error of measurement
TES: Thoracic erector spinae

## References

[1] Reeves N.P., Narendra K.S., Cholewicki J. (2007). Spine stability: The six blind men and the elephant. Clin. Biomech..

[2] Panjabi M.M. (1992). The stabilising system of the spine—Part 1: Function, dysfunction, adaptation and enhancement. J. Spinal Disord..

[3] Panjabi M.M. (1992). The stabilising system of the spine—Part 2: Neutral zone and instability hypothesis. J. Spinal Disord..

[4] Sanchez-Zuriaga D., Lopez-Pascual J., Garrido-Jaen D., Garcia-Mas M.A. (2015). A comparison of lumbopelvic motion patterns and erector spinae behavior between asymtomatic subjects and patients with recurrent low back pain during pain-free periods. J. Manip. Physiol. Ther..

[5] Kim M.H., Yoo W.G., Choi B.R. (2013). Differences between two subgroups of low back pain patients in lumbopelvic rotation and symmetry in the erector spinae and hamstring muscles during trunk flexion when standing. J. Electromyogr. Kinesiol..

[6] Claus A.P., Hides J.A., Moseley G.L., Hodges P.W. (2009). Different ways to balance the spine. Spine.

[7] Hashemirad F., Talebian S., Hatef B., Kahlaee A.H. (2009). The relationship between flexibility and EMG activity pattern of the erector spinae muscles during trunk flexion-extension. J. Electromyogr. Kinesiol..

[8] Burnett A.F., Cornelius M.W., Dankaerts W., O’Sullivan P.B. (2004). Spinal kinematics and trunk muscle activity in cyclists: A comparison between healthy controls and non-specific chronic low back pain subjects—A pilot investigation. Man. Ther..

[9] McGill S.M., Cholewicki J., Peach J.P. (1997). Methodological considerations for using inductive sensors (3SPACE ISOTRAK) to monitor 3-D orthopaedic joint motion. Clin. Biomech..

[10] Kaigle A.M., Wesberg P., Hansson T.H. (1998). Muscular and kinematic behavior of the lumbar spine during flexion-extension. J. Spinal Disord..

[11] Callaghan J.P., Gunning J.L., McGill S.M. (1998). The relationship between lumbar spine load and muscle actitivity during extensor exercises. Phys. Ther..

[12] Peach J.P., Sutarno C.G., McGill S.M. (1998). Three-dimensional kinematics and trunk muscle myoelectric activity in the young lumbar spine: A database. Arch. Phys. Med. Rehabil..

[13] Dankaerts W., O’Sullivan P.B., Burnett A.F., Straker L.M., Davey P., Gupta R. (2009). Discriminating healthy controls and two clinical subgroups of nonspecific chronic low back pain patients using trunk muscle activation and lumbosacral kinematics of postures and movements. Spine.

[14] Breen A.C., Teyhan D.S., Mellor F.E., Breen A.C., Wong K.W.N., Deitz A. (2012). Measurement of intervertebral motion using quantitative fluoroscopy: Report of an international forum and proposal for use in the assessment of degenerative disc disease in the lumbar spine. Adv. Orthop..

[15] D’hooge R., Hodges P., Tsao H., Hall L., MacDonald D., Danneels D. (2013). Altered trunk muscle coordination during rapid trunk flexion in people in remission of recurrent low back pain. J. Electromyogr. Kinesiol..

[16] Hodges P., van den Hoorn W., Dawson A., Cholewicki J. (2009). Changes in the mechanical properties of the trunk in low back pain may be associated with recurrence. J. Biomech..

[17] Luhring S., Schinkel-Ivy A., Drake J.D.M. (2015). Evaluation of the lumbar kinematic measures that most consistently characterize lumbar muscle activation patterns during trunk flexion: A cross-sectional study. J. Manip. Physiol. Ther..

[18] McGorry R.W., Lin J.H. (2012). Flexion relaxation and its relation to pain and function over the duration of a back pain episode. PLoS ONE.

[19] Bogduk N. (2012). Clinical and Radiological Anatomy of the Lumbar Spine.

[20] Okawa A., Shiomiya K., Komori H., Muneta T., Arai Y., Nakai O. (1998). Dynamic motion study of the whole lumbar spine by videofluoroscopy. Spine.

[21] Teyhen D.S., Flynn T.W., Childs J.D., Abraham L.D. (2007). Arthrokinematics in a subgroup of patients likely to benefit from a lumbar stabilization exercise program. Phys. Ther..

[22] Mellor F.E., Thomas P., Thompson P., Breen A.C. (2014). Proportional lumbar spine inter-vertebral motion patterns: A comparison of patients with chronic non-specific low back pain and healthy controls. Eur. Spine J..

[23] Pearson A.M., Spratt K.F., Genuario J., McGough W., Kosman K., Lurie J., Sengupta D.K. (2011). Precision of lumbar intervertebral measurements. Spine.

[24] Teyhen D.S., Flynn T.W., Bovik A.C., Abraham L.D. (2005). A new technique for digital fluoroscopic video assessment of sagittal plane lumbar spine motion. Spine.

[25] Yeager M.S., Cook D.J., Cheng B.C. (2014). Reliability of computer-assisted lumbar intervertebral measurement using a novel vertebral motion analysis system. Spine J..

[26] Knutson L.M., Soderberg G.L., Ballantyne B.T., Clarke W.R. (1994). A study of various normalization procedures for within day electromyographic data. J. Electromyogr. Kinesiol..

[27] Lehman G.J., McGill S.M. (1999). The importance of normalization in the interpretation of surface electromyography: A proof of principle. J. Manip. Physiol. Ther..

[28] Dvorak J., Panjabi M.M., Chang D.G., Theiler R., Grob D. (1991). Functional radiographic diagnosis of the lumbar spine. Flexion-extension and lateral bending. Spine.

[29] Nelson-Wong E., Callaghan J.P. (2010). Is muscle co-activation a predisposing factor for low back pain development during standing? A multifactorial approach for early identification of at-risk individuals. J. Electromyogr. Kinesiol..

[30] O’Shaughnessy J., Roy J.F., Descarreaux M. (2013). Changes in flexion-relaxation phenomenon and lumbo-pelvic kinematics following lumbar disc replacement surgery. J. Neuroeng. Rehabil..

[31] Anatomy.TV. https://anatomy.tv/new_home.aspx?startapp=&startres=&startstudyguide=&S=&ReturnUrl=&lpuserid=&.

[32] Kim H.W., Ko Y.J., Rhee W.I., Lee J.S., Lim J.E., Lee S.J., Im S., Lee J.I. (2007). Interexaminer reliability and accuracy of posterior superior iliac spine and iliac crest palpation for spinal level estimations. J. Manip. Physiol. Therap..

[33] Chin K.R., Kuntz A.F., Bohlman H.H., Emery S.E. (2006). Changes in the iliac crest-lumbar relationship from standing to prone. Spine J..

[34] Billis E.V., Foster N.E., Wright C.C. (2003). Reproducibility and repeatability: Errors of three groups of physiotherapists in locating spinal levels by palpation. Man. Ther..

[35] Chakraverty R., Pynsent P., Isaacs K. (2007). Which spinal levels are identified by palpation of the iliac crests and the posterior superior iliac spines?. J. Anat..

[36] Merz O., Wolf U., Robert M., Gesing V., Rominger M. (2013). Validity of palpation techniques for the identification of the spinous process L5. Man. Ther..

[37] Demoulin C., Vanderthommen M., Duysens C., Crielaard J.M. (2006). Spinal muscle evaluation using the Sorensen test: A critical appraisal of the literature. Joint Bone Spine.

[38] Van Dieen J.H., Cholewicki J., Radebold A. (2003). Trunk muscle recruitment patterns in patients with low back pain enhance the stability of the lumbar spine. Spine.

[39] Reeves N.P., Cholewicki J., Silfies S.P. (2006). Muscle activation imbalance and low-back injury in varsity athletes. J. Electromyogr. Kinesiol..

[40] Shrout P.E. (1998). Measurement reliability and agreement in psychiatry. Stat. Methods Med. Res..

[41] De Vet H.C.W., Terwee C.B., Knol D.L., Bouter L.M. (2006). When to use agreement *versus* reliability measures. J. Clin. Epidemiol..

[42] Mellor F.E. (2014). An Evaluation of Passive Recumbent Quantitative Fluoroscopy to Measure Mid-Lumbar Intervertebral Motion in Patients with Chronic Non-Specific Low Back Pain and Healthy Volunteers. Ph.D. Thesis.

[43] Soderberg G.L., Knutson L.M. (2000). A guide for use and interpretation of kinesiologic electromyographic data. Phys. Ther..

[44] Geisser M.E., Ranavaya M., Haig A.J., Roth R.S., Zucker R., Ambroz C., Caruso M. (2005). A meta-analytic review of surface electromyography among persons with low back pain and normal, healthy controls. J. Pain.

[45] Van Dieen J.H., Selen L.P.J., Cholewicki J. (2003). Trunk muscle activation in low-back pain patients, an analysis of the literature. J. Electromyogr. Kinesiol..

[46] Lariviere C., Arsenault A.B. (2008). On the use of EMG-ratios to assess the coordination of back muscles. Clin. Biomech..

[47] Panjabi M.M., Krag M.H., Dimnet J.C., Walter S.D., Brand R.A. (1984). Thoracic spine centres of rotation in the sagittal plane. J. Orthop. Res..

[48] Kaigle A.M., Holm S.H., Hansson T.H. (1995). Experimental instability in the lumbar spine. Spine.

[49] Kanayama M., Abumi K., Kaneda K., Tadano S., Ukai T. (1996). Phase lag of the intersegmental motion in flexion-extension of the lumbar and lumbosacral spine: An *in vivo* study. Spine.

[50] Bergmark A. (1989). Stability of the lumbar spine: A study in mechanical engineering. Acta Orthop. Scand..

[51] Crisco J.J., Panjabi M.M. (1991). The intersegmental and multisegmental muscles of the lumbar spine: A biomechanical model comparing lateral stabilizing potential. Spine.

[52] Cholewicki J., van Vliet J.J. (2002). Relative contribution of trunk muscles to the stability of the lumbar spine during isometric exertions. Clin. Biomech..

[53] Pavlova A.V., Cooper K., Meakin J.R., Barr R., Aspden R. Internal lumbar spine motion during lifting. Proceedings of the 21st Congress of the European Society of Biomechanics.

[54] Cholewicki J., McGill S.M. (1996). Mechanical stability of the *in vivo* lumbar spine: Implications for injury and chronic low back pain. Clin. Biomech..

[55] Van den Hoorn W., Bruijn S.M., Meijer O.G., Hodges P.W., van Dieen J.H. (2012). Mechanical coupling between transverse plane pelvis and thorax rotations during gait is higher in people with low back pain. J. Biomech..

[56] Willigenburg N.W., Kingma I., van Dieen J.H. (2013). Center of pressure trajectories, trunk kinematics and trunk muscle activation during unstable sitting in low back pain patients. Gait Posture.

[57] Pavlova A.V., Meakin J.R., Cooper K., Barr R.J., Aspden R.M. (2014). The lumbar spine has an intrinsic shape specific to each individual that remains a characteristic throughout flexion and extension. Eur. Spine J..

[58] McGill S.M., Grenier S., Kavcic N., Cholewicki J. (2003). Coordination of muscle activity to assure stability of the lumbar spine. J. Electromyogr. Kinesiol..

